# E4orf1 Improves Lipid and Glucose Metabolism in Hepatocytes: A Template to Improve Steatosis & Hyperglycemia

**DOI:** 10.1371/journal.pone.0047813

**Published:** 2012-10-23

**Authors:** Emily J. Dhurandhar, Rashmi Krishnapuram, Vijay Hegde, Olga Dubuisson, Rongya Tao, X. Charlie Dong, Jianping Ye, Nikhil V. Dhurandhar

**Affiliations:** 1 Pennington Biomedical Research Center, Louisiana State University System, Baton Rouge, Louisiana, United States of America; 2 Department of Biochemistry and Molecular Biology, Indiana University School of Medicine, Indianapolis, Indiana, United States of America; Imperial College London, United Kingdom

## Abstract

Hepatic steatosis often accompanies obesity and insulin resistance. The cornerstones of steatosis treatment include reducing body weight and dietary fat intake, which are marginally successful over the long term. Ad36, a human adenovirus, may offer a template to overcome these limitations. In vitro and in vivo studies collectively indicate that via its E4orf1 protein, Ad36 improves hyperglycemia, and attenuates hepatic steatosis, despite a high fat diet and without weight loss. Considering that hepatic insulin sensitivity, or the synthesis, oxidation, or export of fatty acid by hepatocytes are the key determinant of hepatic lipid storage, we determined the role of E4orf1 protein in modulating these physiological pathways. For this study, HepG2 cells, or mouse primary hepatocytes were transfected with E4orf1 or the null vector. Glucose output by hepatocytes was determined under gluconeogenic conditions (cAMP and dexamethasone, or glucagon exposure). Also, de-novo lipogenesis, palmitate oxidation, and lipid export as determined by apoB secretion were measured 48 h post transfection. Results show that compared to null vector transfected cells, E4orf1 significantly reduced glucose output in basal and gluconeogenic conditions. E4orf1 reduced de-novo lipogenesis by about 35%, increased complete fatty acid oxidation 2-fold (p<0.0001), and apoB secretion 1.5 fold(p<0.003). Response of key signaling molecules to E4orf1 transfection was in agreement with these findings. Thus, E4orf1 offers a valuable template to exogenously modulate hepatic glucose and lipid metabolism. Elucidating the underlying molecular mechanism may help develop therapeutic approaches for treating diabetes or non-alcoholic fatty liver disease(NAFLD).

## Introduction

Impaired hepatic fatty acid oxidation, decreased hepatic lipid export, and increased hepatic de-novo lipogenesis (DNL), are key determinants of hepatic lipid accumulation, which may contribute to non-alcoholic fatty liver disease (NAFLD) [1], [2], [3], [4]. Fatty liver is also associated with impaired insulin action in liver, skeletal muscle, and adipose tissue [5], [6], [7], [8], [9], [10]. In the presence of type 2 diabetes mellitus (T2DM), individuals with NAFLD have a greater risk of progressing to cirrhosis [11] or non-alcoholic steatohepatitis (NASH) [12]. NASH is a more advanced stage of NAFLD, which includes inflammation and fibrosis in addition to lipid accumulation that is thought to further contribute to hepatic insulin resistance.

Effective control of hepatic steatosis may improve insulin resistance and attenuate the progression to NAFLD or more serious complications. Weight loss and lowering dietary fat intake are the cornerstones of treatment for steatosis, yet these interventions are marginally effective in the long term [13]. Bariatric surgery is effective for weight loss, but it is highly invasive and expensive and limited to selected volunteers who meet the rigorous screening criteria. Several drugs are under investigation for treating NAFLD, but none is approved by the FDA for this purpose and results have been variable [14]. Therefore, novel drugs are urgently needed for the treatment of NAFLD.

Interestingly, human Adenovirus Ad36 offers a novel template for discovery of therapeutic agents that may attenuate NAFLD and NASH. Ad36 infection improves glycemic control in mice, and prevents hepatic steatosis even in the presence of a high fat diet [15]. Ad36 activates a Ras-mediated phosphoinositide-3-kinase (PI3K) activation pathway to increase Glut4 and glucose clearance in adipose tissue and skeletal muscle [15], [16], [17]. Ad36 DNA and RNA is present in livers of infected mice, and the amount of viral DNA in the liver is correlated with improvement in blood glucose levels [15]. *In vivo* protein and gene expression data indicate that Ad36 infection suppresses hepatic glucose output and hepatic inflammation, and increases hepatic fatty acid oxidation and lipid export [15]- all of which may contribute to reduced hepatic steatosis, reduced hepatic insulin resistance, and improved whole body glucose disposal. Additionally, this is a phenomenon relevant to humans, since previous Ad36 infection in adults and children is associated with improved glycemic control [15] and lower liver fat accumulation and prevalence of NAFLD [18], [19]. Thus, Ad36 appears to influence glucose and hepatic lipid homeostasis.

It would be valuable to elucidate how Ad36, an exogenous factor, favorably modulates hepatic metabolism. Also, it is unknown if the improvement in liver metabolism is a direct effect of Ad36 or a secondary effect of Ad36-mediated metabolic changes. Considering the significant number of viral proteins produced during active Ad36 replication, it is challenging to elucidate the precise mechanism. E4orf1 protein of Ad36 was recently identified as the protein likely to mediate Ad36 induced improvements in glucose disposal [20]. E4orf1 is necessary and sufficient for Ad36-induced glucose uptake in preadipocytes, adipocytes, and myoblasts, and it effectively suppresses glucose release from hepatocytes [20]. Reducing uncontrolled hepatic glucose output is a key approach in improving glycemic control. A single agent that blocks hepatic glucose output and simultaneously improves hepatic insulin resistance and hepatic steatosis, was recently reported [21]. Similarly, we hypothesized that in hepatocytes, E4orf1 may reduce glucose output even in the presence of gluconeogenic conditions, and simultaneously reduce lipid accumulation.

To investigate this hypothesis, we transfected HepG2 cells and primary mouse hepatocytes with an E4orf1 containing plasmid, to determine glucose output in gluconeogenic conditions, and to measure synthesis, oxidation, and export of fatty acids. The set of experiments described below tested a) if the protective effect of Ad36 on hepatic metabolism could be ascribed to its E4orf1 protein and b) whether E4orf1 could directly influence hepatocytes. We expected that E4orf1 may provide an excellent template to develop novel drugs for the treatment of NAFLD or T2DM, if indeed it favorably modulated glucose and lipid homeostasis in liver cells.

**Figure 1:**
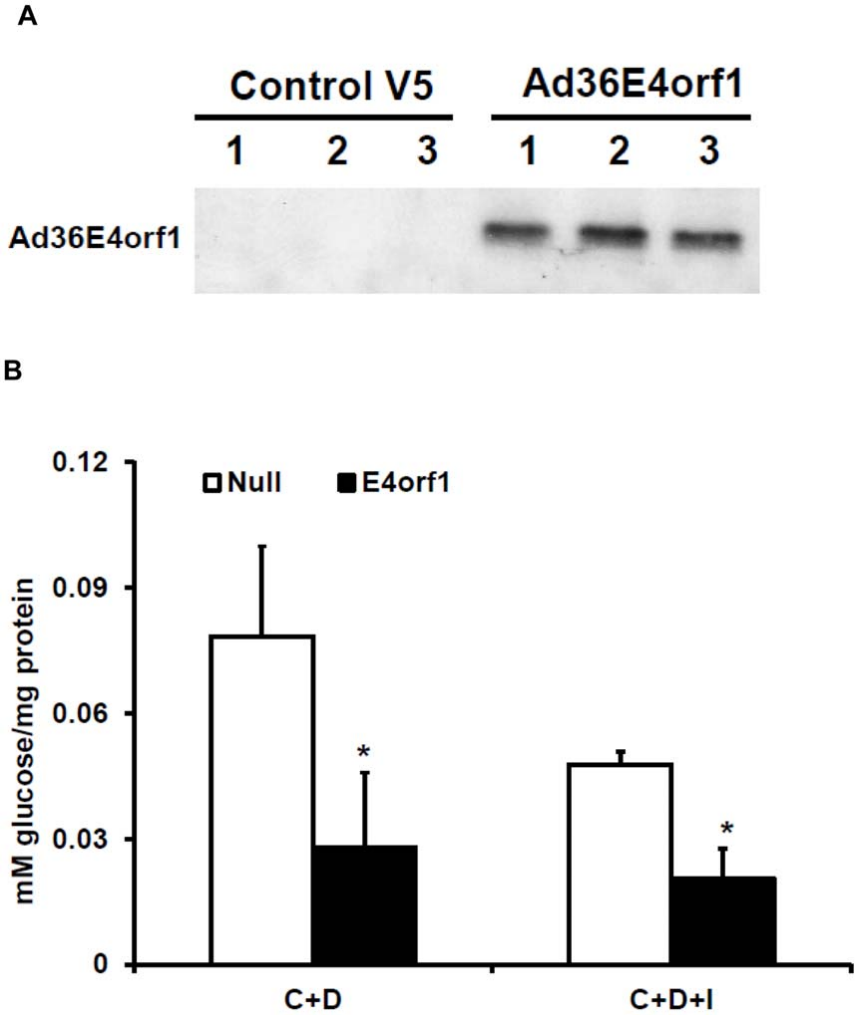
E4orf1 suppresses glucose output in the presence of gluconeogenic stimulators in HepG2. (A) Western blot showing Ad36 E4orf1 protein expression in HepG2 cells following transfection with V5-Ad36 E4orf1 plasmid DNA. (B) Glucose output by HepG2 cells following E4orf1 or null Vector transfection. Cells were stimulated with gluconeogenic cAMP (1 mM) (C) and Dexamethasone (500 nM) (D) in the absence or presence of insulin (I). Expressed as mean mM glucose per mg of protein, ± SD. E4orf1 significantly suppresses glucose output in the basal and insulin stimulated conditions (p = 0.0001 and 0.003, respectively).

## Materials and Methods

Each experiment is described in detail below, followed by detailed protocols in the Techniques and Assays (T&A) section.

### A. Experiments

#### Experiment 1. Glucose output: To determine the effect of E4orf1 on hepatocyte glucose output in gluconeogenic conditions

HepG2 cells were transfected with a plasmid expressing V5 tagged E4orf1 (V5-E4orf1) or null plasmids via electroporation (described in T&A). Two days later, output of glucose in media was determined under the following conditions. After 4 hours of serum starvation, cells were rinsed with PBS and treated with cAMP (1 mM) and Dexamethasone (500 nM) as gluconeogenic stimulators, with 0 or 10 nM insulin for 3 hours in glucose free media. A glucose oxidase assay determined media glucose concentrations, which were normalized to well-protein content. Eight biological replicates were tested per group. Proteins were harvested for determining Ad36 E4orf1 expression along with Ras and Glut2 abundance by western blotting.

**Figure 2:**
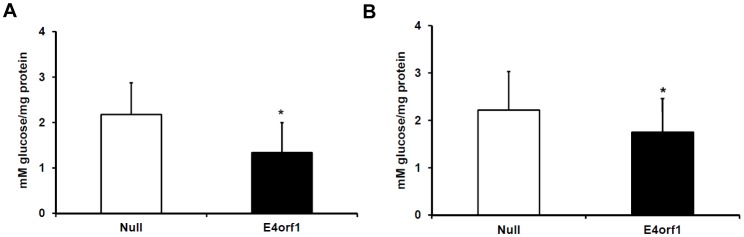
Glucose Output from Primary Hepatocytes Transfected with E4orf1. Glucose output in mouse primary hepatocytes after transfection with E4orf1 or null vector. A) Glucose output by primary hepatocytes after serum and glucose starvation for 1.5 hours, expressed as mean mM glucose per mg protein ± SD. E4orf1suppressed glucose output compared to Null vector (p = 0.0001). B) Glucose output in primary hepatocytes treated with 100 nM glucagon in serum-free, and glucose-free media for 1.5 hr. E4orf1 suppressed glucose output in this condition (p = 0.029). Expressed as mean mM glucose per mg protein, ± SD.

**Figure 3:**
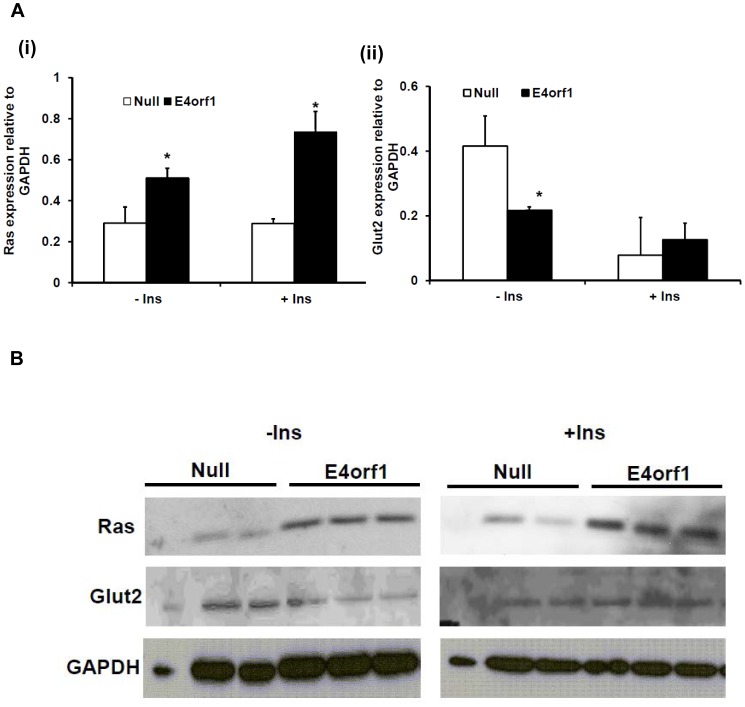
E4orf1 transfection increases Ras abundance and suppresses Glut2 levels in HepG2. A) Densitometry for Ras and Glut2 expression normalized to GAPDH. E4orf1 transfected cells have (i.) Significantly greater Ras expression in basal and insulin stimulated condition (p = 0.001 and 0.004, respectively) and (ii.) Significantly lower Glut2 levels in the basal condition (p = 0.04). B) Western blots of Ras and Glut2 expression in HepG2.

Next, primary hepatocytes obtained from 129Sv, and C57BL/6J mixed background mice were assayed for glucose output in serum free, glucose free condition as described above. Two days post transfection of primary hepatocytes with V5-E4orf1 or null plasmids, glucose output was determined in presence of 0 or 100 nM glucagon. A preliminary experiment with 8 replicates was conducted to standardize experimental conditions, followed by a final experiment where 24 replicates per group were tested.

**Figure 4:**
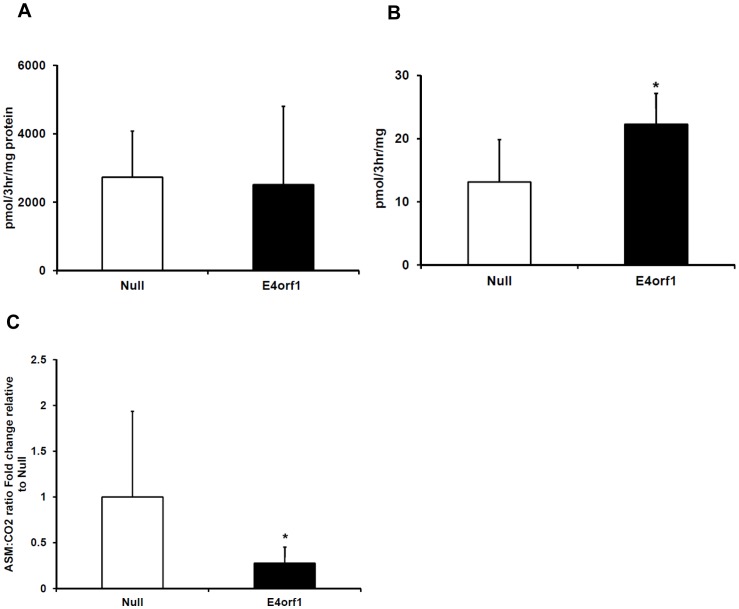
[^14^C]-Palmitate oxidation after transfection of E4orf1 in HepG2. A) Total palmitate oxidation (complete + partial), expressed as pmol palmitate/3hr/mg protein, Mean ± SD. No significant differences. B). Complete palmitate oxidation, expressed as pmol palmitate/3hr/mg protein mean ± SD. E4orf1 transfection significantly increased complete oxidation of palmitate compared to Null (p = 0.004). C) Ratio of acid soluble metabolites (incomplete oxidation) to CO_2_ (complete oxidation), expressed as mean fold change relative to null + SD. E4orf1 has significantly lower ratios compared to Null.

#### Experiment 2. Fatty acid oxidation: To determine the effect of E4orf1 on fatty acid oxidation in HepG2 cells

To determine the effect of E4orf1 on fatty acid oxidation, two separate experiments were conducted. Initially, PolyJet (SignaGen #SL100688) was used to transfect HepG2 cells with V5- E4orf1 or null plasmids as described in T&A. A palmitate oxidation assay was conducted two days after transfection as described in T&A. Eight biological replicates per group were tested for total and complete palmitate oxidation, which was normalized to protein content.

**Figure 5:**
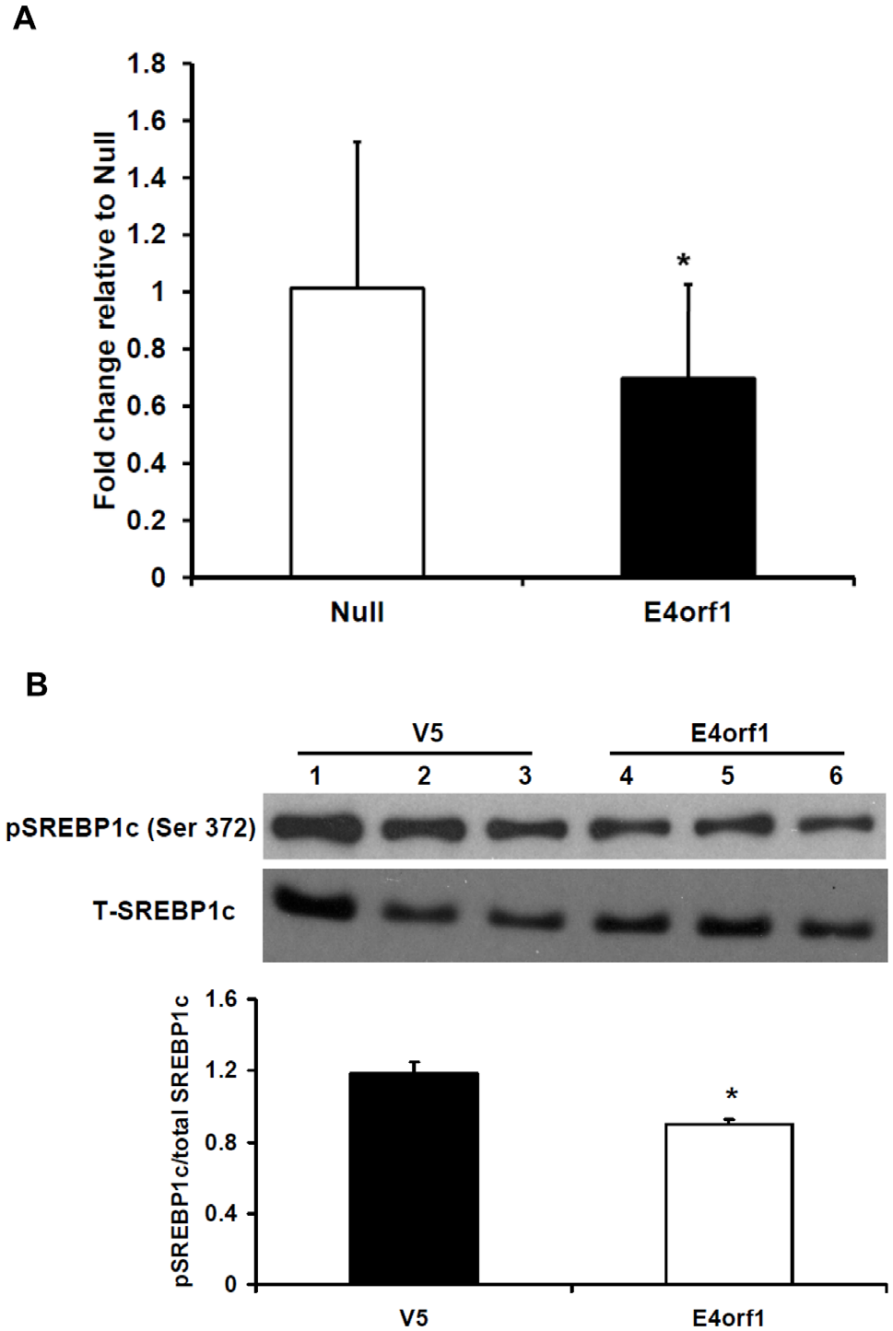
DNL in HepG2 following E4orf1 transfection. HepG2 cells were transfected with E4orf1 or null vector. A) Expressed as mean fold change in pmol [^14^-C]-glucose/mg protein relative to null, ± SD. Two separate experiments were combined. E4orf1 significantly decreases DNL compared to null (p = 0.01). B) E4orf1 significantly decreases phosphorylated SREBP1c expression compared to null (V5) (p = 0.001) indicating reduced DNL in these cells.

A second palmitate oxidation assay was conducted to verify the changes in ratio of complete to partial fatty acid oxidation by E4orf1. Experimental conditions remained the same as described above and included eight biological replicates. The percent partial oxidation or the ratio of partial to complete oxidation were expressed as a fold change compared to that in the respective null vector transfected groups in the two experiments.

**Figure 6:**
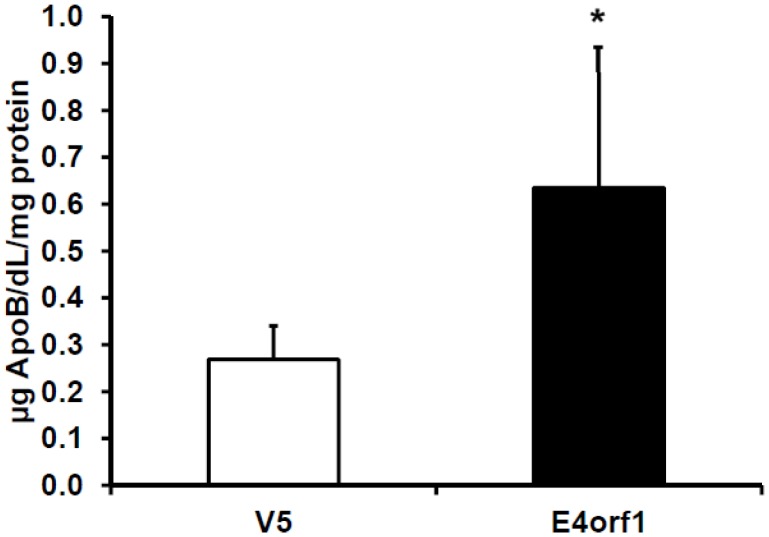
ApoB Secretion from HepG2 Transfected with E4orf1. ApoB secretion in conditioned media from HepG2 transfected with E4orf1 or null vector. Expressed as mean µg ApoB/dL/mg protein, ± SD. Ad36 E4orf1 increased ApoB secretion into media compared to the null vector (V5) (p = 0.01).

#### Experiment 3. Denovo lipogenesis: To determine the effect of E4orf1 on hepatocyte denovo lipogenesis

HepG2 cells were transfected with V5- E4orf1 or null plasmids with PolyJet as described in T&A. Two days post transfection, a DNL assay was conducted as described in T&A. The experiment was repeated twice, each time with eight biological replicates per group, and the results were expressed as fold change relative to the null transfected group. Protein lysates extracted from the transfected HepG2 cells were used to determine phosphorylated SREBP-1c (*sterol regulatory element-binding protein-1c)* abundance –a key regulator of denovo lipogenesis. Three biological replicates were used per group.

#### Experiment 4. Lipid export: To determine the effect of E4orf1 on hepatocyte ApoB secretion

HepG2 cells were transfected with V5- E4orf1or null plasmids with PolyJet, as described in T&A. The media was collected 48 hours post transfection to determine ApoB concentration. About 24 hours post transfection, cells were switched to Dulbecco’s minimum essential media (DMEM) without phenol red (Invitrogen # 31053–036) plus 1.5% bovine serum albumin (BSA) and incubated for another 24 hours. The media samples were collected and stored at −20°C. ApoB secretion was determined via ELISA (Alerchek # A70102) according to the manufacturer’s instructions except the modification where 1∶50 dilution of media was used instead of the suggested 1∶1000 dilution. ApoB secretion was normalized to protein content of each well. Eight biological replicates per group were tested.

### B. Techniques & Assays

#### *a.* Cell Culture

HepG2 cells were obtained from Dr. Jianping Ye at the Pennington Biomedical Research Center, LA. They were maintained in high glucose DMEM, 10% fetal calf serum, and an antibiotic-antimycotic agent (1%).

Primary mouse hepatocytes were obtained from Dr. Charlie Dong, Indiana University School of Medicine, Indianapolis, IN. They were maintained in William’s Medium E (Invitrogen # 12551-032) supplemented with 2 mM glutamate, 10% FBS, and primocin (Invivogen # ant-pm-1). To obtain primary hepatocytes, all animal procedures were performed following the guidelines of the National Institutes of Health Guide for the Care and Use of Laboratory Animals, and studies were approved by the Indiana University School of Medicine Animal Care and Use Committee.

#### *b.* Palmitate Oxidation Assay

Cells were incubated in 500 µL high glucose DMEM (Invitrogen # 11995081) supplemented with 100 µM palmitate (Sigma Aldrich, #P9767-10G), 1 µCi/mL [^14^C]-Palmitate (PerkinElmer # NET043001MC), 1 mM carnitine (Sigma Aldrich #C0158), 0.25% bovine serum albumin (Sigma Aldrich # A7030), and 12.5 mM HEPES buffer for 3 hours. During the incubation, plates were covered with parafilm to minimize CO_2_ loss. After incubation, 400 µL of media was transferred to a CO_2_ trapper, adjacent to a separate well containing 200 µL of 1 M NaOH such that gas exchange could occur between the two wells. After loading samples and closing the trapper from further gas exchange using a silicone cover, 40 µL of 70% perchloric acid (Sigma Aldrich # 311421) was injected into the well containing media. After rotating the CO_2_ trapper for 1 hour, all 200 µL of [^14^C]-CO_2_– containing NaOH was removed and placed in 5 mL scintillation fluid. Once media was collected, cells were solubilized in 0.05% sodium dodecyl sulfate (SDS). A BCA (bicinchoninic acid) assay was used to measure the protein content. After incubation, the perchloric acid treated media was collected, stored at 4°C overnight, and centrifuged at 4°C for 10 minutes at 15,000 g. Acid-soluble metabolites were detected by placing 200 µL of the supernatant in 5 ml scintillation fluid, and the samples were read on a Beckman scintillation counter. For calculations, original media with isotope and a vial containing only scintillation fluid for background subtraction was read. The total activity was calculated as nmol palmitate oxidized in 3 h per mg of protein.

#### c. Denovo Lipogenesis Assay

Cells were serum starved for 1 h in glucose free DMEM (Invitrogen #, 11966025) then incubated with 500 µL high glucose DMEM (Invitrogen # 11995081) with 1 µCi/mL [^14^C]-Glucose (PerkinElmer #NEC042A001MC) for 3 hours. Cells were treated with the same [^14^C]-palmitate media used for the palmitate oxidation assay for 3 hours. After treatment, media was discarded and cells washed in ice-cold PBS. Two hundred µL 0.05% SDS was used to lyse cells and cell lysates collected. Samples were centrifuged for 5 min, at 15,000 g, at 4°C to clear them of lysate debris and 20 µL of sample collected for protein determination. The pellet was then resuspended, and 1 mL of chloroform:methanol (2∶1) was added to each sample. The samples were rocked at room temperature for 15 minutes, and 0.5 mL double distilled water was then added to the samples. Samples were rocked again for 15 minutes, followed by centrifugation for 10 min at 3,000 rpm, at 4°C. The lower organic phase was collected in scintillation vials and read on a Beckman scintillation counter. Results were normalized to protein content.

#### d. Plasmid Preparation and Transfection

HepG2 cells were transfected using the AMAXA Nucleofector II device, Cell Line Nucleofector Kit V (Lonza #VACA-1003) with either pcDNA-V5-E4orf1(E4orf1) or pcDNA-V5-DEST (Null) plasmids. Two µg of plasmid was used for 2×10^6^ cells per cuvette, and Nucleofector program T-028 was used for transfection. After transfection, cells were resuspended and 40,000 cells per well were plated in 96-well plates for the glucose output assay. Transfection was verified via WB for the detection of V5-tagged E4orf1 with V5 antibodies.

For HepG2 lipid oxidation, DNL, and ApoB secretion studies, PolyJet (SignaGen #SL100688) was used. For each well, 1.5 µg plasmid and 4.5 µL Polyjet were complexed, and transfection complex was left on cells overnight for 14–16 hours.

Primary mouse hepatocytes were transfected with the AMAXA Nucleofector II device, Mouse Hepatocyte Nucleofector Kit (Lonza # VPL-1004). Six µg of either pcDNA-V5-DEST (Null) plasmids or pcDNA-V5-E4orf1 (E4orf1), 7×10^5^ cells per cuvette, and Nucleofector program T-028 were used for transfection, and after transfection cell were resuspended and 40,000 cells per well were plated in 96-well plates for the glucose output assay. Cell culture medium was replaced with fresh media 4 hours post transfection.

#### e. Glucose Oxidase Assay

A colorimetric assay was used to determine glucose output into media (Raichem, #R80038). A standard curve diluted in serum free, glucose free media was used to determine the concentration of glucose in unknown samples. The standard curve was created by 5X serial dilutions of 5 µM glucose solution, such that the curve ranged from 5–0.04 µM. Ten µL of media was used for each unknown sample. After collecting the media, HepG2 cells were solubilized in 0.05% SDS to determine the protein concentration by BCA (Sigma Aldrich # B9643, #C2284). Primary hepatocytes, were solubilized in RIPA buffer after several rapid freeze-thaw cycles. Each sample was centrifuged at 14,000 rpm, at 4°C for 10 minutes, and protein concentration in the supernatant was determined. Glucose in the media was normalized to protein content of the well.

#### f. Western Blotting

Cells were harvested in RIPA buffer supplemented with anti-protease (Sigma Aldrich, #P8340) and anti-phosphatase inhibitor cocktail (Thermo Scientific #78420). Protein concentration was determined by BCA assay. SDS-PAGE was performed with 15 µg protein loaded on a 10% gel and proteins were transferred to PVDF membrane. Ad36 E4orf1 expression was determined by using 30 µg protein lysate and detected by a custom made antibody at a dilution of 1∶1000 (Proteintech group Inc, IL). For detecting Ras (Cell Signaling #3965S), a 1∶1,000 dilution of antibody was used. For detection of Glut2, 10 µg protein was loaded, and a 1∶200 dilution of primary antibody was used (Santa Cruz # sc-7580). GAPDH was used as a loading control, which was detected with 1∶1000 dilution of antibody (Cell Signaling #2118). Total SREBP1c and phospho-SREBP1c were detected by immunoblotting 30 g protein separated on a 8% gel by SDS-PAGE with 1∶1000 dilution of respective antibodies (SREBP1c (Ser372) was a gift from Dr. Jianxin Xie, (Development Scientist, Cell Signaling Technology, Boston, MA).

### C. Statistics

All metabolic assays were conducted with at least 8 biological replicates, and one-sided student’s t-test was used to determine significant differences. All data were inspected for normality of distribution and homogeneity of variance to ensure assumptions of student’s t-test were valid. If variances were not equal, Welch’s test was used instead of student’s t-test. HepG2 glucose output, fat oxidation, and denovo lipogenesis assays were repeated twice. All metabolic assays were normalized to protein content. For the ApoB ELISA, two technical replicates were used for each sample.

For western blot analyses, three biological replicates were tested for each group. All proteins were normalized to a loading control protein. Student’s t-test was used to determine significant differences between groups.

## Results

### Experiment 1. Glucose output

The liver is a major organ in the control of blood glucose through production of glucose. Increased hepatic glucose output is observed in obesity and is a mechanism of hyperglycemia. This study determined the glucose output by a human hepatocyte cell line HepG2, in response to E4orf1 protein. Firstly we determined Ad36 E4orf1 protein expression in these cells following transfection by western blotting (Figure 1A). Interestingly, E4orf1 suppressed glucose output from HepG2 cells in the presence of cAMP and dexamethasone (gluconeogenic stimulators), in basal and insulin treated conditions (p = 0.00001 and 0.003, respectively) (Figure 1B). The experiment was also conducted in mouse primary hepatocytes, and E4orf1 suppressed glucose output compared to the null vector (p = 0.0001) (Figure 2A). The hormone glucagon is a powerful inducer of glucose production in hepatocytes. Even in the presence of 100 nM glucagon, Ad36 E4orf1 was sufficient to suppress glucose output from primary hepatocytes (p = 0.02) (Figure 2B). ). Although the variance in the primary cell glucose output was high and there was significant overlap between the distributions of the two groups, the means were still significantly different. To understand the mechanism, we examined cellular signaling in hepatocytes. E4orf1 transfection increased basal and insulin stimulated Ras abundance (p = 0.001 and 0.004, respectively) and suppressed Glut2 levels under basal condition (p = 0.04) in HepG2 cells (Figure 3), suggesting E4orf1 may suppress glucose output by reducing Glut2 abundance.

### Experiment 2. Fatty Acid oxidation

We examined fatty acid catabolism in hepatocytes with radio-labeled palmitate. Complete and partial [^14^C]- palmitate oxidation were measured after transfection with Ad36 E4orf1or null plasmid. Total (complete + partial) oxidation was not significantly different between the null and E4orf1 transfected cells (Figure 4A), however, completely oxidized palmitate was significantly higher in E4orf1 transfected cells (p = 0.004) (Figure 4B). E4orf1 significantly decreased the ratio of incomplete to complete oxidation (p = 0.008) (Figure 4C). The variance in the ratio of incomplete to complete oxidation was high particularly in the null transfected cells, and although the overlap between the distributions is high, the means were significantly different.

### Experiment 3. Denovo Lipogenesis (DNL)

Ad36 E4orf1 significantly suppressed DNL in HepG2 cells (p = 0.01) (Figure 5A). Protein abundance and phosphorylation of SREBP1c were determined as key indicators of DNL enzyme activity. There was no significant difference in SREBP1c abundance between E4orf1 transfected and null cells. However, the ratio between total and phosphorylated SREBP1c showed a significant reduction in phospho-SREBP1c in E4orf1 transfected cells (Figure 5B), indicating reduced activation of SREBP1c [22].

### Experiment 4

Lipid export by hepatocytes is indicated by ApoB-containing lipoprotein secretion into the media. Ad36 E4orf up-regulated ApoB secretion into media compared to null vector transfected cells (p = 0.01) (Figure 6).

## Discussion

Previous animal studies showed that Ad36 improves systemic glycemic control and attenuates hepatic lipid accumulation even in presence of a high fat diet. Several alterations in hepatic lipid metabolism can result in insulin resistance and hepatic steatosis such as impaired suppression of glucose output under gluconeogenic conditions, impaired fatty acid oxidation, decreased lipid export, and increased DNL, although the latter two are the two major contributors to NAFLD [1], [2], [3]. In vitro studies showed that E4orf1 protein is necessary and sufficient for Ad36 to increase cellular glucose uptake from adipocytes and myoblasts and to reduce glucose output from hepatocytes. Therefore, we tested the effect of E4orf1 on key factors that may influence hepatic insulin sensitivity and lipid accumulation. Results presented herein suggest E4orf1 is a promising template for improving NAFLD and glycemic control.

We previously reported that E4orf1 is sufficient to suppress glucose output in the serum-free, and glucose-free conditions. The suppression of glucose output by E4orf1 is even more robust than that induced by 10 nM insulin [20], suggesting suppression of hepatic glucose output may be one mechanism for Ad36 infection-induced improvements in glycemic control. Here we show that even in gluconeogenic conditions, E4orf1 is sufficient to suppress glucose output in HepG2 and primary mouse hepatocytes. As previously demonstrated in 3T3-L1 preadipocytes and adipocytes [20], E4orf1 increases Ras abundance in HepG2 cells, which may contribute to the observed reduction in Glut2 abundance, as previously reported in a pancreatic beta cell model [23]. Thus E4orf1 may act through a similar mechanism to reduce Glut2 levels and glucose output.

Although E4orf1 failed to up-regulate total fatty acid oxidation, it increased complete oxidation of fatty acids to CO_2_ and decreased the ratio of partial to complete oxidation. Perhaps, in presence of E4orf1, fatty acids that enter the beta oxidation pathway are more likely to be completely oxidized. E4orf1 therefore may prevent buildup of fatty acid intermediates and attenuate subsequent inhibition of hepatic insulin signaling [24]. Importantly, E4orf1 also reduced DNL, and up-regulated ApoB secretion, which is indicative of lipid export, in HepG2 cells. These two pathways are integral to the development of NAFLD in response to a high fat diet, so this action of E4orf1 may be sufficient to attenuate hepatic triglyceride accumulation, as observed in Ad36 infected C57BL/6J mice on high fat diet [15].

It was recently demonstrated that hepatic glucose output, lipid accumulation, and insulin resistance could be reduced simultaneously by knock down of cAMP response element binding protein [21]. Similarly, E4orf1 can perform these functions simultaneously. However, at this time it is unclear if this is through a single or multiple pathways. Importantly, it was determined that E4orf1 has a marked direct effect on hepatic lipid metabolism; this suggests that presence of E4orf1 in the liver alone may be sufficient to improve steatosis. Adiponectin secretion from adipose tissue induced by Ad36 E4orf1 [15], [16] may, however, add to these benefits *in vivo*, and produce a more pronounced effect on fatty acid oxidation, than that observed in hepatocytes *in vitro*.

There are some limitations of this study. All conclusions are based on *in vitro* studies and need to be verified *in vivo* in an appropriate animal model. E4orf1 is not a secretory protein, and therefore does not have a cell surface receptor for cell entry. Moreover, a long term delivery of E4orf1 may be required to influence metabolism in vivo. Therefore, a suitable delivery system such as a retrovirus or nano-particles that express E4orf1 is required to test the effects of E4orf1 in vivo. Another approach could be to develop chemical analogs that mimic the action of E4orf1. Regardless, the metabolic effects of E4orf1 are promising, and may provide novel signaling targets to prevent NAFLD and insulin resistance even in the presence of a high fat diet.
